# Metabolic profiling reveals potential metabolic markers associated with Hypoxia Inducible Factor-mediated signalling in hypoxic cancer cells

**DOI:** 10.1038/srep15649

**Published:** 2015-10-28

**Authors:** Emily G. Armitage, Helen L. Kotze, J. William Allwood, Warwick B. Dunn, Royston Goodacre, Kaye J. Williams

**Affiliations:** 1 School of Chemical Engineering and Analytical Science, Manchester Institute of Biotechnology, University of Manchester, Manchester M1 7DN, UK; 2 School of Chemistry, Manchester Institute of Biotechnology, University of Manchester, Manchester M1 7DN, UK; 3 Centre for Endocrinology and Diabetes, Institute of Human Development, The University of Manchester, Manchester M13 9PL, UK; 4 Centre for Advanced Discovery and Experimental Therapeutics (CADET), Central Manchester University Hospitals NHS Foundation Trust, York Place, off Oxford Road, Manchester M13 9WL, UK; 5 Manchester Pharmacy School, University of Manchester, Oxford Road, Manchester M13 9PT, UK

## Abstract

Hypoxia inducible factors (HIFs) plays an important role in oxygen compromised environments and therefore in tumour survival. In this research, metabolomics has been applied to study HIFs metabolic function in two cell models: mouse hepatocellular carcinoma and human colon carcinoma, whereby the metabolism has been profiled for a range of oxygen potentials. Wild type cells have been compared to cells deficient in HIF signalling to reveal its effect on cellular metabolism under normal oxygen conditions as well as low oxygen, hypoxic and anoxic environments. Characteristic responses to hypoxia that were conserved across both cell models involved the anti-correlation between 2-hydroxyglutarate, 2-oxoglutarate, fructose, hexadecanoic acid, hypotaurine, pyruvate and octadecenoic acid with 4-hydroxyproline, aspartate, cysteine, glutamine, lysine, malate and pyroglutamate. Further to this, network-based correlation analysis revealed HIF specific pathway responses to each oxygen condition that were also conserved between cell models. From this, 4-hydroxyproline was revealed as a regulating hub in low oxygen survival of WT cells while fructose appeared to be in HIF deficient cells. Pathways surrounding these hubs were built from the direct connections of correlated metabolites that look beyond traditional pathways in order to understand the mechanism of HIF response to low oxygen environments.

Hypoxia inducible factors are heterodimeric transcription factors that can promote survival in low oxygen environments and are prevalent in solid tumours. The most studied is HIF-1 which is known to regulate a multitude of genes and proteins. HIF-2 can similarly influence gene and protein expression however the effects of HIF-mediated signalling on the metabolome, the functional level many consider to be most closely related to the phenotype, are less well characterised. HIFs consist of two subunits: the constitutively expressed β subunit and the oxygen labile α subunits that are subject to post translational modification and subsequent degradation in the presence of oxygen^1^. Under low oxygen conditions, degradation is inhibited and the α subunits translocate to the nucleus in order to complex with HIF-1β^2^. The activated complex controls the regulation of genes containing hypoxia response elements (100–200 genes)^3^.

HIF-1 is known to up-regulate genes encoding glucose transporters (Glut-1 and 3) in addition to a range of other glycolytic enzymes, including those controlling the rate of glycolytic flux (hexokinase, phosphofructokinase, pyruvate kinase and lactate dehydrogenase (LDH)^4^. HIF-1 also induces expression of monocarboxylate transporter-4 (MCT-4), which facilitates the metabolic co-cooperativity between aerobic and hypoxic cells^5^, whereby hypoxic cells generate and excrete lactate *via* up-regulated LDH and MCT-1 activity and the aerobic tumours cells utilise the lactate metabolism following uptake via MCT-1^5^.

Both tumour suppressor inactivation and oncogene activation can contribute to HIF activity and have consequential effects on metabolism^4^. Lack of HIF-1 β subunit expression and consequential prevention of heterodimer formation and HIF function causes phenotypic alterations particularly with respect to metabolism^6^. For example, ATP content can be compromised by up to 80% with HIF-1 β deficiency. Most research concerning HIFs and its interaction with the metabolome has focused on glycolysis. Revealing the effects of HIF on the metabolome as a whole system has the potential to reveal metabolic pathways beyond glycolysis that are important in cancer phenotype and that are potential targets for cancer therapy.

The use of metabolomics based approaches in cancer research is growing and a range of applications in this field have been recently reviewed^7^. In this research metabolic profiling has been employed to discover HIF dependent (and independent) influences on regulation of the global metabolome in cancer by identifying metabolic signatures representative of HIF function in oxygen deprived environments. Intracellular extracts from cells without HIF function have been compared to wild type (WT) cell extracts (i) to observe which features are missing when HIF is not functional, (ii) to discover features that are added in low oxygen environments with a lack of HIF function and (iii) to discover features independent of HIF-function.

Three oxygen conditions have been compared (normoxia-21%, hypoxia-1% and anoxia-0%) in order to study the phenotypic responses of cells with and without functional HIF. Although the oxygen conditions selected in this study are used by many to imitate normoxia, hypoxia and anoxia^8^, an initial study was undertaken to evaluate whether atmospheric (21%) oxygen is reflective in metabolic terms of exposure to 5% oxygen which may be considered to be closer to the physiological level. Fourier transform infra-red (FT-IR) spectroscopy was used as a global metabolic fingerprinting method to confirm 21% oxygen as a valid condition to represent normoxia.

Following the determination of oxygen conditions, two models were used to investigate the impact of HIF-function on the metabolome under oxygen deprived conditions: human colorectal HCT 116 and murine hepatoma HEPA-1 cells and derivatives lacking HIF-function *via* expression of a dominant negative HIF-1α construct (HCT116 DN) or through lack of HIF-1β (HEPA-1 c4). Metabolic profiles of WT and HIF deficient cells have been analysed using gas chromatography-mass spectrometry (GC-MS). Human and murine models were employed in order to reveal conserved and differential effects of HIF-function on the cellular metabolome. Multi-level analysis of this data including univariate, multivariate and network-based correlation analysis has provided mechanistic insight into the role of HIF in global metabolism. For example, key metabolic hubs for WT and HIF deficient cells have been revealed allowing identification of potentially important enzyme activities driving HIF-dependent metabolic effects. The determination of hubs and key pathways that change in response to HIF function or oxygen treatment provides insight into the cellular response to hypoxic stress and potentially reveals targets for cancer therapy.

## Results

### FT-IR fingerprint validation of oxygen conditions

**Figure 1:**
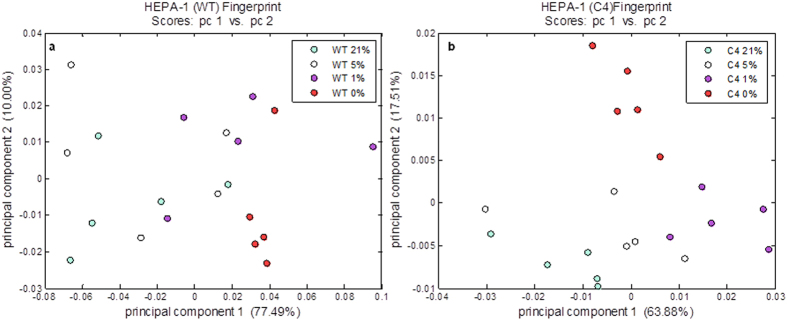
Principal components analysis (PCA) scores plots of PC 1 *versus* PC 2 for intracellular FT-IR metabolic fingerprints of HEPA-1 wild type (WT) (left) and C4 (right) cells exposed to 21% oxygen, 1%, 5% and 0% oxygen. In both cell lines, intracellular profiles from 21% and 5% oxygen group and separate from 1% and 0% highlighting the similarity between 21% and 5%.

FT-IR spectroscopy was employed as a rapid screening method to evaluate whether the cellular metabolome observed under ambient atmospheric oxygen levels of 21% is reflective of that observed at more physiological levels of 5% oxygen and whether this was distinct from metabolic characteristics under oxygen deprivation. FT-IR spectroscopy was used to compare the intracellular fingerprints of HEPA-1 WT and C4 cells cultured at 21%, 5%, 1% or 0%. Details of the methodology for this are given in supplementary information. Figure 1 shows the scores plots of principal component (PC) 1 *versus* PC 2 for HEPA-1 WT and C4 cells; where in both cases there is a grouping of 21% and 5% samples that separate from the lower oxygen samples. This indicated that there was no significant difference between the profiles of cells cultured at 21% compared to 5% supporting that either could be used to represent the normoxic condition and validating the use of 21% oxygen in subsequent metabolic fingerprinting experiments.

### Metabolic profiling

Metabolic profiling utilising GC-MS was performed to determine the effect of HIF-function on the metabolism of cells exposed to different oxygen conditions (normoxia: 21%, hypoxia: 1%, anoxia 0%). From the analysis of both WT and HIF deficient cells, a total of 42 and 41 peaks passed our protocol tests and had relative standard deviations (RSDs) lower than 30% in the GC-MS profiling of QC samples in the HCT 116 and HEPA-1 models respectively. These were identified to MSI guidelines as (referred to in methods) and are listed in Table 1 of supplementary information. Heat maps of peak intensities were generated for each model and are shown in Fig. 2 of supplementary information. Peak numbers are in accordance to the order in CVA and in table 1 of the supplementary information. Additionally, data are supplied as supplementary information.

**Figure 2:**
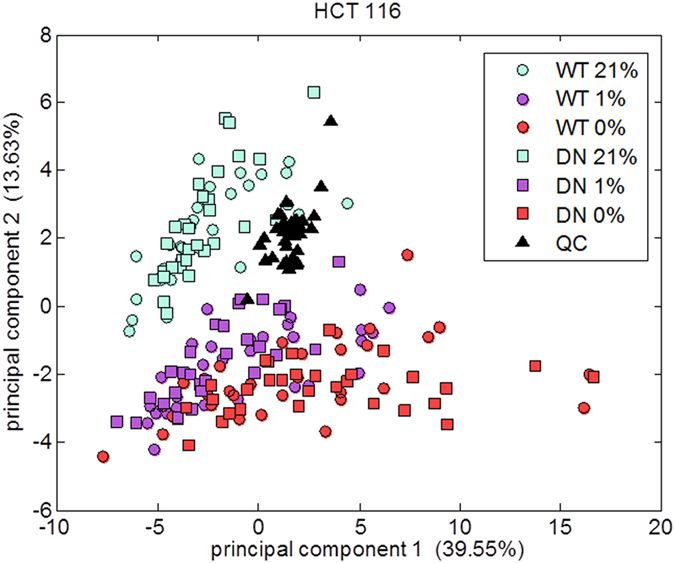
Principal components analysis (PCA) on all HCT 116 metabolic profiles analysed using gas chromatography-mass spectrometry (GC-MS). The quality control (QC) samples formed from pooling small quantities from each sample displayed on the plot fall approximately in the middle. WT = wild type; DN = dominant negative.

An initial PCA was performed on all samples from each cell line including QC samples to confirm that the batch matching process was successful. The plot generated from this analysis of the HCT 116 cell line is shown in Fig. 2, where the QC samples were observed to fall in the centre of the PCA plot and amongst other data points suggesting the data were correctly pre-processed. This plot also shows that the variation in metabolism is greater affected by oxygen level than by HIF: it is not possible to separate WT and DN cells by PCA.

**Figure 3:**
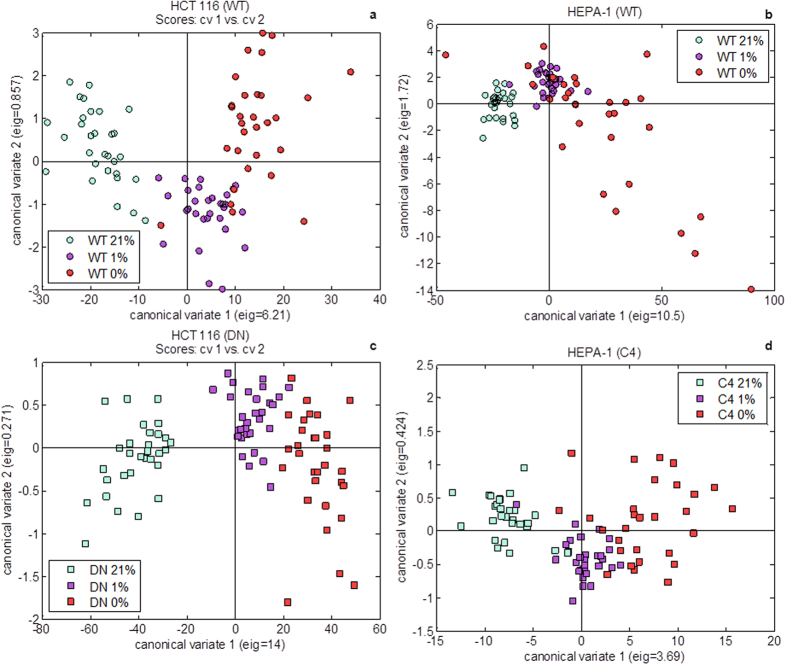
Canonical variates analysis (CVA) scores plots for (a) HCT 116 wild type (WT), (b) HEPA-1 WT, (c) HCT 116 dominant negative (DN) and (d) HEPA-1 C4 samples comparing all 3 oxygen conditions. Each CVA model was built using 8 principal components (PCs) collectively accounting for between 80% and 90% of the total explained variance from each analysis. In all cases the greatest separation in the data was between normoxic samples and low oxygen samples in canonical variate (CV) 1.

Canonical variates analysis (CVA) was performed in order to identify individually how WT and HIF deficient cells respond to the range of oxygen treatments. This was conducted separately for HCT 116 WT samples, HCT 116 DN samples, HEPA-1 WT samples and HEPA-1 C4 samples. In all cases a gradient between oxygen tensions could be identified with a clear separation in CV 1 between normoxic samples and low oxygen samples (Fig. 3). These separations were caused by different anti-correlations between metabolite features that appeared to be controlled by HIF. The loadings from these analyses were assessed in turn to identify features of normoxia and low oxygen samples in each plot which were then compared to each other to find similarities as a function of oxygen exposure and as a function of HIF presence or absence in each cell model. The results from this are summarised in Table 1.

**Figure 4:**
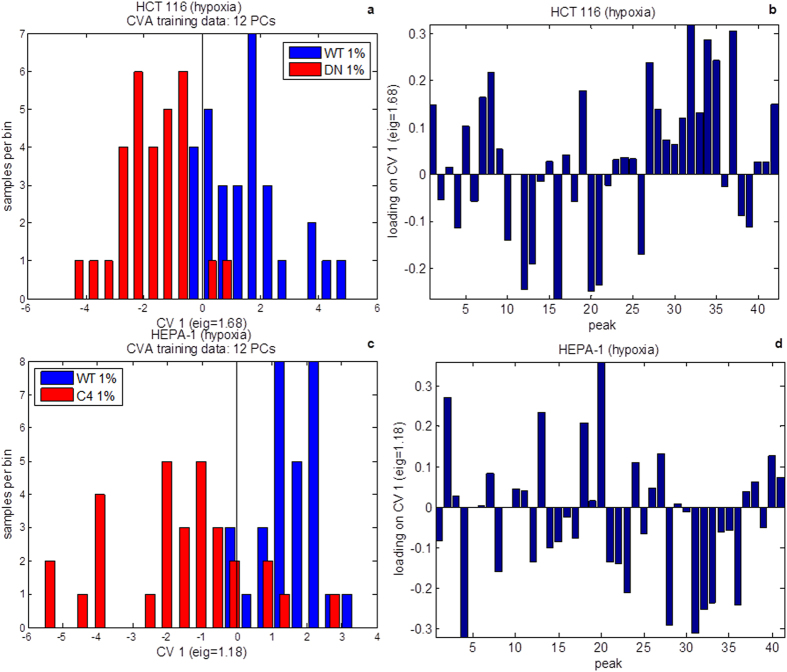
Canonical variates analysis (CVA) of hypoxic (1% oxygen) samples in the HCT 116 (a,b) and HEPA-1 (**c**,**d**) cell models. In each case the CVA models were built using principal components (PCs) 1–12 accounting for approximately 90% or the total explained variance. The distributions of samples in canonical variate (CV) 1 for each cell model are represented as bar charts (**a**,**c**) where the number of samples per bin is shown. The loadings for CV 1 for each cell model are shown (**b**,**d**). The peak number refers to unique metabolites and follows the same order as in Table 1 of supplementary information.

A direct comparison between cells with and without functional HIF was made by performing CVA on hypoxic WT and DN cells then on WT and C4 cells. It was hoped that understanding the multi-component variation in the metabolome as a response to hypoxia with respect to HIF activity would lead to elucidating the role of HIF in cancer cell metabolism, especially as there are oxygen gradients within solid tumour. Furthermore, comparing HCT 116 and HEPA-1 analyses identified the conserved responses of WT and HIF deficiency. The results from this CVA are shown in Fig. 4.

### Network-based correlation analysis of metabolites

**Figure 5:**
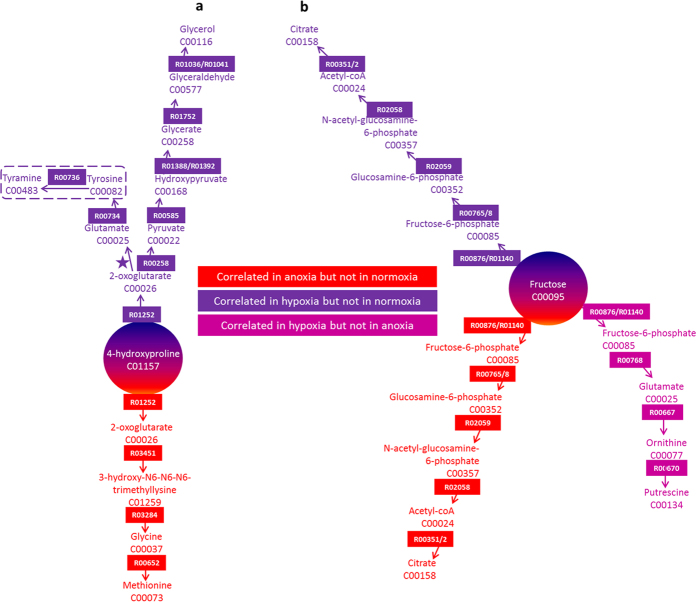
Network-based correlation analysis for HCT 116 and HEPA-1 cell models. Conserved correlation differences between normoxia, hypoxia and anoxia were mapped onto the EHMN reconstruction using a shortest path analysis described **previously**^13^. Pathways are coloured according to which oxygen levels correlation differences occurred and are presented for a) WT cells and b) HIF-1 deficient cells of both models. KEGG compound and reaction identifiers are indicated. Tyrosine and tyramine are contained in a box to denote that this metabolite was not definitively identified in the GC-MS analysis.

Correlation analysis was performed metabolite-by-metabolite in a pair-wise fashion and significant differences in correlation coefficients were identified in the following comparisons for each cell model: normoxia *versus* hypoxia, normoxia *versus* anoxia and hypoxia *vs*. anoxia. Following this, comparisons were made between models to identify conserved correlations of HIF-1 activity in both cell models. Since the two cell models were of different species origin, these correlations are likely to be the most conserved and potentially the key regulators of HIF associated metabolism. In the case of HIF deficient cells, they represent the most conserved regulations as compensation to low oxygen survival when HIF is not functional. In both HCT 116 and HEPA-1 models, there were two conserved correlation differences in WT cells between normoxia and hypoxia: between 4-hydroxyproline and glycerol and between 4-hydroxyproline and tyrosine/tyramine. In HIF deficient cells, there was one correlation difference between normoxia and hypoxia: between fructose and citrate. In both HCT 116 and HEPA-1 models, there was one conserved correlation difference in WT cells between normoxia and anoxia: between 4-hydroxyproline and methionine. In HIF deficient cells, there was also one correlation difference between normoxia and anoxia: between fructose and citrate. There were no correlation differences between hypoxia and anoxia in WT cells, however in HIF deficient cells there were two: between fructose and glutamate and between fructose and putrescine. The shortest paths between differently correlated metabolites were calculated and these pathways are depicted in Fig. 5.

## Discussion

Intracellular extracts from cells without HIF function have been compared to WT cell extracts (i) to observe which features are lost when HIF is not functional, (ii) to discover features that are added in low oxygen environments with a lack of HIF function and (iii) to discover features independent of HIF-function. Cancer therapy based on perturbing pathways associated with identified metabolites will require both the knowledge of how the system functions in the presence of HIF and the mechanisms employed in the absence of HIF. Aside from revealing potential metabolic targets for future cancer therapy, revealing markers of HIF metabolism could lead to a better understanding of the phenotype and may enable more successful diagnosis and prognosis of cancer in patients. Screening tumour extracts for relevant signatures determined for each oxygen level in these *in vitro* experiments could help identify the level of hypoxia in the tumour. The present experiments were not designed to identify the relative contributions of HIF-1 or 2 to metabolic phenotype. From previous work HIF-1 appears the predominant driver of hypoxia-mediated responses in both models used in the current study (unpublished observations). However the nature of the modifications within the derivative cells renders both HIF-1 and 2 non-functional giving a more global impact of HIF-signalling on metabolic regulation.

CVA revealed metabolic signatures that could be attributed to cellular response to lowering oxygen, seemingly controlled or not by HIF and signatures that revealed the same phenotypic responses, irrespective of cell line. From Table 1, it is possible to subdivide metabolites into four types of metabolic signatures: features of oxygen treatment irrespective of HIF and conserved between cell lines; features of oxygen treatment irrespective of HIF but dependent on cell line; features that were HIF specific but cell line dependent and finally features that were HIF specific in both cell lines. Many responses were conserved between HCT 116 and HEPA-1 cells which provided confidence in recognising the results as HIF related metabolites.

**Figure 6:**
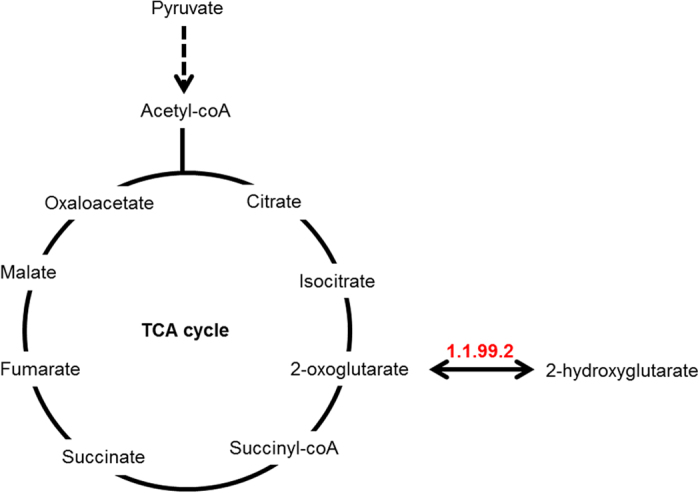
The interaction between pyruvate, 2-oxoglutarate and 2-hydroxyglutarate and central carbon metabolism. 2-hydroxyglutarate dehydrogenase (KEGG enzyme 1.1.99.2) catalyses a reversible reaction between 2-oxoglutarate and 2-hydroxyglutarate. All 3 metabolites feed into the tricarboxylic acid (TCA) cycle.

The direct comparison between cells with and without functional HIF made by performing CVA on hypoxic WT and DN cells or WT and C4 cells for HCT 116 and HEPA-1 cell models respectively enabled evaluation of the impact of HIF on metabolism in hypoxia. Although the loadings (Fig. 4b,d) demonstrated considerable variance between the two cell models, there were similarities in the anti-correlation between metabolites causing the separation between WT and HIF deficient cells in each. The positive loadings and features of WT cells for each cell line consisted of 2-hydroxyglutarate, 2-oxoglutarate, fructose, hexadecanoic acid, hypotaurine, pyruvate and octadecenoic acid which were anti-correlated with 4-hydroxyproline, aspartate, cysteine, glutamine, lysine, malate and pyroglutamate (which is a cyclisation product of glutamate during the chemical derivatisation process) in the negative loadings. It is likely that 2-hydroxyglutarate, 2-oxoglutarate, fructose, hexadecanoic acid, hypotaurine, pyruvate and octadecenoic acid are associated with HIF signalling and that these targets are conserved across species. In addition to pyruvate, 2-oxoglutarate and 2-hydroxyglutarate are all connected to central carbon metabolism *via* the TCA cycle. Figure 6 shows a schematic for how these three metabolites interact. Since they are affected by the presence or absence of HIF, this provides some evidence that HIF targets central carbon metabolism, more specifically through the replenishment of TCA intermediates through the process of anaplerosis.

It is of interest that 2-hydroxyglutarate and 2-oxoglutarate are elevated in a HIF-dependent fashion. 2-hydroxyglutarate is commonly described as an “onco-metabolite” through apparent correlations of enhanced abundance and tumourigenecity. Much emphasis has been placed on generation of 2-hydroxyglutarate via a mutated form of isocitrate dehydrogenase-1 (IDH1-R132X) which is a common feature of gliomas^9^. However the HCT 116 model used in these studies has WT IDH1^10^. As highlighted in Fig. 6, this perhaps implicates 2-hydroxyglutarate dehydrogenase (KEGG enzyme code 1.1.99.2)^11^. This could be via direct or indirect mechanisms. 2-hydroxyglutarate has been implicated as an inhibitor of the prolyl hydroxylase (PHD) enzymes that are required for degradation of the HIF-α subunits whilst 2-oxoglutarate is required for PHD activity. Modifications in the relative balance of the two could conceivably modify HIF-signalling. Further complexity arises with the recent observation of non-enzymatic oxidation of 2-hydroxyglutarate to 2-oxoglutarate^12^. Although the physiological relevance of this process is yet to be proven, it is likely that both enzymatic and non-enzymatic reactions contribute to overall metabolite balance which can potentially both influence HIF-function and be influenced by the metabolic environment promoted by HIF. Two very recently published articles, confirm our observations that hypoxia induces cellular production of 2 hydroxyglutarate^13^,^14^. Mechanisticially, 2-hydroxyglutarate production appeared dependent on promiscuous substrate use by LDH-A. The authors found HIF to be sufficient but not necessary for 2-hydroxyglutarate production. This observation aligns with how dependent LDH-A expression is on HIF-activity within the cell models used. LDH-A is expressed in a HIF-dependent manner in both of the models used in our studies (Hepa-1WT and c4 data published in Golinska *et al.* 2011^15^, HCT 116 WT and DN unpublished observations, Cowen RL and Williams KJ). This is exploited in the HIF-activity reporter system we use (driven by an LDH-A-promoter) and explains the HIF-dependency of 2-hydroxyglutarate production in these model systems.

**Figure 7:**
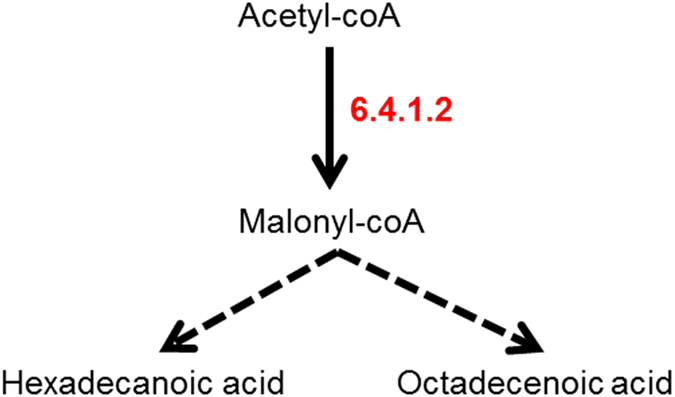
Fatty acid (FA) biosynthesis. Hexadecanoic acid and octadecenoic acid are two of the end points of FA biosynthesis that metabolises acetyl-coA *via* malonyl-coA. Acetyl-CoA carboxylase alpha (KEGG enzyme 6.4.1.2).

Hexadecanoic acid and octadecenoic acid, found in WT cells, are both features of multiple metabolic pathways including fatty acid (FA) biosynthesis (KEGG pathway ko00061). Figure 7 shows a schematic for FA biosynthesis where hexadecanoic acid and octadecenoic acid are endpoints. FA biosynthesis was the other main pathway highlighted by comparing WT and HIF deficient cells, suggesting a role for HIF in regulating this metabolic pathway.

It is clear from CVA that the mechanisms of cellular response to hypoxia and the role HIF plays in this response is complex. Although some relationships could be made to existing pathways it is difficult to interpret metabolomics results on this level. Network-based correlation analysis offered a more complete insight into the cellular responses to lowering oxygen that were different in the case of HIF functionality or deficiency. Using network-based correlation analysis reveals potential pathway targets that are constructed not from the traditional schematics of pathways but from the direct connections between metabolites that can cross many 'traditional' pathway boundaries^4^,^16^. Further to revealing new target pathways, this method allows the clear identification of 'hub' metabolites that appear to play a central role in the control of cellular response to lowering oxygen. From the current studies, when HIF is functional, 4-hydroxyproline appears as a hub metabolite, while fructose appears as a hub metabolite when HIF is deficient. In the case of 4-hydroxyproline, its associated pathways may be a feature of survival in hypoxia or anoxia relative to normoxia. The correlation between fructose and citrate appeared to be of importance in low oxygen survival (correlated in hypoxia and anoxia relative to normoxia), however when comparing hypoxia and anoxia, a different pathway between fructose and putrescine was highlighted. Figure 5 shows the pathway responses to low oxygen surrounding these hubs. Irrespective of oxygen level, deprivation, KEGG reaction R01252 is the key responding reaction that involves proline hydroxylase (KEGG code 1.14.11.2), the activity of which could be positively influenced by the 2-hydroxyglutarate/2-oxoglutarate changes also observed in WT cells. In contrast the key reactions surrounding fructose as the hub metabolite in HIF deficient cells are R00876 and R01140. These reactions involve hexokinase (2.7.1.1), isoform 2 of which (HK2) is strongly implicated in driving high glycolytic flux, ribonucleotide synthesis via the pentose phosphate pathway and maintenance of TCA intermediates via anaplerosis^17^, consistent with the metabolite and network correlations observed. Paradoxically both HK1 and HK2 are recognised transcriptional targets of HIF-1^18^,^19^, yet here, in the absence of HIF activity, a specific reliance on this enzyme is revealed.

In conclusion, a range of metabolites have been discovered from GC-MS analysis of WT and HIF-deficient HCT 116 and HEPA-1 cells exposed to differing levels of oxygenation that reflect both HIF dependent and independent processes often conserved across species.

Some of the most interesting HIF-related metabolites discovered from multivariate analysis using CVA were 2-hydroxyglutarate, 2-oxoglutarate, hexadecanoic acid, hypotaurine, pyruvate and octadecenoic acid. These findings warrant investigation of the role of HIF in hypotaurine metabolism, butanoate metabolism, FA biosynthesis and biosynthesis of unsaturated FAs. Network-based correlation analysis revealed 4-hydroxyproline to be a regulated hub in low oxygen survival of WT cells of both models while fructose appeared to be a hub in HIF deficient cells. This analytical method also offered a complementary way of revealing a mechanistic basis of how HIF function influences cancer metabolism.

## Materials and Methods

### Cell lines

HEPA-1 WT, HIF-1β-deficient HEPA-1 C4, HCT 116 WT and HCT 116 cells expressing a dominant negative (DN) HIF-1α construct are described in previous publications: development and validation of HEPA-1 C4 is given in Hoffman *et al.* (1991)^20^, Maxwell *et al.* (1997)^21^ and Troy *et al.* (2005)^6^, where HIF-1β was found not to be expressed in these cells. The HIF-1α DN construct is detailed in Brown *et al.* (2006)^22^ and the HCT116-DN model in Roberts *et al.* (2009)^8^.

### Experimental conditions for growth

For each sample obtained for metabolic profiling by GC-MS, cells were seeded in exponential phase at 1 × 10^6^ cells/mL in RPMI 1640 (Gibco BRL, Paisley, UK) supplemented with 10% undefined foetal calf serum (FCS) (Labtech International, East Sussex, UK) and 2 mM glutamine (Sigma-Aldrich, Dorset, UK) into a 10 cm^2^ Petri dish (Falcon, Runcorn, UK). They were maintained in normoxia (95% air and 5% CO_2_ at 37 °C and 95% relative humidity) for 24 h in the conditions described for routine culture. For exposure to 24 h oxygen treatments, samples were divided into three groups: 30 replicates to remain in normoxia, 30 replicates to be exposed to hypoxia (1% oxygen) and 30 replicates to anoxia (0% oxygen). The hypoxic condition was achieved using a closed vessel though which gas containing 1% O_2_, 5% CO_2_ balanced with N_2_ was flowed^23^. The anoxic condition utilised a gloved chamber (Bactron anaerobic chamber, Sheldon Manufacturing, Cornelius, Oregon, USA) in which all parameters other than oxygen were comparable to the other two conditions and all necessary sample preparation and incubation was done in this system. Residual oxygen was removed by flowing supply gas over a palladium catalyst. Cell harvesting in each experiment was conducted in normoxia (for normoxic samples) and in anoxia for hypoxic or anoxic samples whereby the gas taps were locked on the hypoxia chamber after the removal of the hypoxic gas inlet to prevent re-oxygenation of hypoxic samples.

### Validation of oxygen treatments

Oxygen conditions were chosen to be 21% representing normoxia, 1% representing hypoxia and 0% representing anoxia. Fourier transform infrared (FTIR) -spectroscopy was employed as a fingerprinting technique in the selection of oxygen conditions (particularly in the validation of selecting 21% oxygen as the condition to represent normoxia, rather than 5% that could be the more physiologically relevant level, but that was not observed to differ experimentally from 21%). Details of this assay and the results are given in supplementary information.

Samples for metabolic profiling by GC-MS were collected over 4 batches for HCT 116 cells and 4 batches for HEPA-1 cells, where each batch contained an equal number of WT and HIF-1 deficient cells exposed to normoxia, hypoxia or anoxia. In total there were 6 experimental groups per cell model (WT and HIF-1 deficient forms exposed to normoxia, hypoxia or anoxia).

In every batch, the level of HIF-1 transcriptional activity was measured for a sample in each experimental group using a luciferase reporter-based assay^23^. This was performed as a quality check to ensure HIF-1 transcriptional activity consistency between batches of samples collected for GC-MS analysis. In all cases, HIF-1 activity was shown to increase in hypoxic and anoxic WT cells compared to normoxic WT cells that was not the case in HIF-1 deficient cells. The methods and results from this can be found in supplementary information.

### Metabolite extraction

For metabolite extraction, extracellular medium was first decanted and cells were washed three times with 1 mL PBS at room temperature. Immediate quenching of metabolism was achieved by first adding 1 mL methanol (maintained at −48 °C) to each sample. Subsequently, cell scrapers were used to remove the adherent cells from the culture surface and the cell suspension was transferred into 1.5 mL Eppendorf tubes (Fisher Scientific, Loughborough, UK). A series of 3 freeze-thaw cycles using liquid nitrogen was performed for all samples followed by centrifugation at 17000 × *g* for 15 min. Supernatants were transferred into new 1.5 mL Eppendorfs and the volume to be lyophilised was normalised according to the weight of the dry pellet following extraction for intracellular samples (achieved using HETO VR MAXI with RVT 4104 refrigerated vapour trap (Thermo Life Sciences, Basingstoke, UK)). Remaining supernatants after normalisation were used to generate QCs which contained an equal proportion (150 μL) of each sample. Supernatants for both the samples and QCs were lyophilised after the addition of 100 μl of an internal standard (0.18 mg/ml succinic d4 acid in water).

### Sample preparation for GC-MS

Prior to metabolic profiling by GC-MS analysis, all samples were chemically derivatised using a two-stage process: the addition of 50 μL of a 20 mg/mL solution of O-methoxylamine in pyridine followed by vortex mixing and heating at 60 °C for 30 min, and the addition of 50 μL of N-methyl-N-trifluoroacetamide followed by vortex mixing and heating at 60 °C for 30 min. Subsequently, debris was pelleted through centrifugation at 17000 × *g* for 10 min and finally, 20 μL of a retention index marker solution containing 0.3 mg/mL n-decane, n-dodecane and n-pentadecane, n-docosane and n-nonadecane in pyridine was added. The resulting supernatants were analysed by GC-MS.

The GC-MS instrument setup has been previously described^24^, and involves an Agilent 6890 GC (Agilent Technologies, Stockport, U.K.) coupled to a LECO Pegasus III (Leco Corp., St. Joseph, MO) EI-ToF-MS. Data acquisition followed a previously optimised sequence^25^: starting with a derivatisation blank, followed by five QC samples, then by five samples and one QC, repeatedly until the end of the analysis. Samples were injected at volumes of 2 μL and the total analysis duration for a single sample was 25 min. The temperature was 70 °C for 4 min and increased by 20 °C every min until 300 °C, which was subsequently maintained for a further 4 min.

All data were pre-processed using the ChromaTOF v3.25 software package. From a set of samples representative of all sample classes, the mass spectrum and retention index of all unique metabolites were exported to a reference table. This reference table was then used to match metabolites in all samples, where it was reported if a mass spectral match between the reference table and the sample peak was greater than 70% and the retention index deviation between them was less than 10. All data were normalised to the peak area of the internal standard (peak area metabolite/peak area internal standard). These data were exported as ‘.csv’ files for subsequent data analysis.

By matching the mass spectrum and retention index to those present in an in-house mass spectral library constructed with data acquired from authentic chemical standards, it was possible to identify a proportion of detected metabolites. A mass spectral match greater than 80% and a retention index match ± 20 provided a definitive identification^26^. For instances where a definitive identification (MSI level 1) to our in-house library could not be made, the mass spectrum was searched against other mass spectral libraries. For example, the National Institute of Standards and Technology (NIST05) database of retention data for non-polar and polar stationary phases^27^ and the publically accessible Golm Metabolome Database of mass spectral libraries^28^ were used to identify metabolites putatively where a match greater than 80% was observed (MSI level 2). The level of identification reported was applied according to reporting guidelines as described by the MSI^29^.

### Data treatment

A previously optimised method for data-pre-processing, involving control of metabolic features with excessive drift in signal^25^, was performed as follows: the RSD of each detected feature was calculated across all QC samples and for features, where in cases that the RSD exceeded 30%, that feature was removed from the whole data set. QCs were also used in signal correction within and between analytical blocks. For this quality control based robust LOESS (locally estimated scatter-plot smoothing) signal correction (QC-RLSC) was applied^25^. This process was performed after feature identification and before analysis of GC-MS data.

Pre-processed data were exported for univariate and multivariate data analysis in Matlab as a data matrix (*m* × *n*) where *m* denoted metabolite features and *n* denoted sample. Values were chromatographic peak areas included for each feature detected in each sample. Prior to data analysis, outliers were identified within each experimental group as values greater than 2.5 standard deviations away from the mean for that metabolite in that group and were subsequently replaced by the mean^30^.

All data analyses were implemented using the Statistics Toolbox in Matlab. Data were observed to fit an approximately normal distribution after log transformation and therefore parametric tests were applied. A two-way analysis of variance (ANOVA) was employed to identify metabolites that differed significantly due to oxygen level, to HIF-1 presence or absence or due to an interaction between oxygen and HIF-1. Since many metabolites were being tested in parallel a false discovery test^31^, using α = 0.1 was applied to control the critical *p-*value for rejecting the null hypothesis that there was no significant difference between oxygen levels and HIF-1 presence or absence. Box plots were generated for significant *p-*values <0.05 that passed the false discovery test.

The first method for multivariate analysis was principal components analysis (PCA). Spectra were scaled by autoscaling^32^. After PCA, multivariate analysis was extended to canonical variates analysis (CVA) whereby PCA was performed and a number of PCs were used along with the experimental group structure to build a model for maximising the between-group variance while minimising the within-group variance. This was performed without exceeding the number of PCs that additively described 95% of the total variance in the data in order to reduce the likelihood of over fitting which can invalidate the model.

Network-based correlation analysis was performed following our recently published method^16^. Briefly, Pearson correlation analysis was performed metabolite-by-metabolite in a pair-wise fashion and significant differences in correlation coefficients were identified in the following comparisons for each cell model; normoxia *versus* hypoxia, normoxia *versus* anoxia and hypoxia *vs*. anoxia. Since the number of samples exceeded 27, the difference in correlation coefficients in each comparison was considered significant when it exceeded 0.407, when at least one correlation had an absolute value <0.7. Finally the shortest paths between differently correlated metabolites were calculated in Matlab using the Edinburgh human metabolic network (EHMN)^33^.

## Additional Information

**How to cite this article**: Armitage, E. G. *et al.* Metabolic profiling reveals potential metabolic markers associated with Hypoxia Inducible Factor-mediated signalling in hypoxic cancer cells. *Sci. Rep.* **5**, 15649; doi: 10.1038/srep15649 (2015).

## Supplementary Material

Supplementary Information

Supplementary Dataset 1

## Figures and Tables

**Table 1:** Comparison of the loadings from canonical variate analysis (CVA) of all 3 oxygen conditions.

Metabolite	HCT 116: loading common between WT and DN	HEPA-1: loading common between WT and C4
4-aminobutyrate	normoxia	
4-hydroxyproline	normoxia	
Allose/Mannose/Galactose /Glucose (1)	*	*
Allose/Mannose/Galactose /Glucose (2)	*	*
Allose/Mannose/Galactose /Glucose (3)		normoxia
2-hydroxyglutarate	low oxygen	
2-oxoglutarate	normoxia*	normoxia*
Aspartate (1)		normoxia
Aspartate (2)	normoxia*	normoxia*
Beta-alanine		normoxia
Citrate	normoxia*	normoxia*
Creatinine		low oxygen
Cysteine	normoxia	
Erythronate /Threonate	low oxygen*	low oxygen*
Fructose (1)	*	*
Fructose (2)	normoxia	
Fructose / Sorbose (1)	low oxygen	
Fructose / Sorbose (2)	low oxygen*	low oxygen*
Glutamate	normoxia	low oxygen
Glutamine	normoxia*	normoxia*
Glycerol	low oxygen*	low oxygen*
Glycerol-3-phosphate		low oxygen
Glycine		low oxygen
Hexadecanoic acid	*	*
Hypotaurine	low oxygen*	low oxygen*
Lactate	low oxygen*	low oxygen*
Leucine		N/A
Lysine	low oxygen	
Malate	normoxia	
Malonate	low oxygen*	low oxygen*
Methionine	low oxygen*	low oxygen*
Norleucine	low oxygen*	low oxygen*
Phosphate	low oxygen*	low oxygen*
Phosphocreatinine	N/A	
Putrescine	normoxia*	normoxia*
Pyroglutamate	normoxia	
Pyruvate		normoxia
*Scyllo*-/*Myo*-inositol	*	*
Octadecenoic acid	*	*
Threonine	low oxygen*	low oxygen*
Tyramine/Tyrosine	low oxygen	
Tyrosine	normoxia*	normoxia*
xylitol/Ribitol	low oxygen	N/A

CVA was performed for HCT 116 wild type (WT) samples, HCT 116 dominant negative (DN) samples HEPA-1 WT samples and HEPA-1 C4 samples (Fig. 3a–d). Metabolites labelled ‘normoxia’ were identified in the loadings as being features of normoxia that were common between WT and HIF-1 deficient cells, while metabolites labelled ‘low oxygen’ were identified in the loadings as being features of low oxygen that were common between WT and hypoxia inducible factor 1 (HIF-1) deficient cells. Un-labelled metabolites represent HIF-1 mediated responses to oxygen since the loadings were not common between WT and HIF-1 deficient cells. Metabolites marked with an asterisk (*) indicate cell line similarities such that the same behaviour was observed in both HCT 116 and HEPA-1cell lines.
