# Supplementary material for: Metabolic profiling reveals potential metabolic markers associated with Hypoxia Inducible Factor-mediated signalling in hypoxic cancer cells
Source: Sci Rep. 2015 Oct 28;5:15649. doi: 10.1038/srep15649 (PMC4623531; doi:10.1038/srep15649)
Supplement: Supplementary Information [file srep15649-s1.pdf]

Metabolic profiling reveals potential metabolic markers associated with Hypoxia Inducible Factor-mediated signalling in hypoxic cancer cells

Emily G. Armitage[1,2], Helen L. Kotze[1], J. William Allwood[3,4], Warwick B. Dunn[3,5,6,7], Royston Goodacre[3], and Kaye J. Williams[8]

1. School of Chemical Engineering and Analytical Science, Manchester Institute of Biotechnology, University of Manchester, Manchester M1 7DN, UK.
2. Present address: Centre for Metabolomics and Bioanalysis (CEMBIO), Facultad de Farmacia, Campus Monteprincipe, Universidad CEU San Pablo, Boadilla del Monte, Madrid, 28668, Spain.
3. School of Chemistry, Manchester Institute of Biotechnology, University of Manchester, Manchester M1 7DN, UK.
4. Present address: Environmental & Biochemical Sciences Group, The James Hutton Institute, Invergowrie, Dundee DD2 5DA, UK
5. Present address: School of Biosciences, University of Birmingham, Edgbaston, Birmingham, B15 2TT, UK.
6. Centre for Endocrinology and Diabetes, Institute of Human Development, The University of Manchester, Manchester M13 9PL, UK.
7. Centre for Advanced Discovery and Experimental Therapeutics (CADET), Central Manchester University Hospitals NHS Foundation Trust, York Place, off Oxford Road, Manchester M13 9WL, UK.
8. Manchester Pharmacy School, University of Manchester, Oxford Road, Manchester M13 9PT, UK.

Supplementary Information

Validation of the oxygen conditions by FT-IR spectroscopy

Cell lysates were prepared for Fourier transform infrared (FT-IR) spectroscopy analysis which was performed to assess the comparison between oxygen treatments. The HEPA-1 model was used to validate the normoxic oxygen treatment of 21 % oxygen which was then subsequently used for metabolic profiling using gas chromatography – mass spectrometry (GC-MS) of all cell lines. The procedure involved seeding cells at 1.5×10^5 in 1.5 mL fresh medium in 3.5 cm cell culture dishes. For each oxygen condition (0 %, 1 %, 5 % and 21 %), 5 biological replicates were prepared for each cell line: WT and C4. All samples were incubated in normoxia for 24 h then transferred to normoxia, hypoxia or anoxia for a further 24 h. The hypoxic condition was achieved using a closed vessel though which gas containing 1% O_2, 5% CO_2 balanced with N_2 was flowed [1]. The anoxic condition utilised a gloved chamber (Bactron anaerobic chamber, Sheldon Manufacturing, Cornelius, Oregon, USA) in

which all parameters other than oxygen were comparable to the other two conditions and all necessary sample preparation and incubation was done within this system. Residual oxygen was removed by flowing supply gas over a palladium catalyst. Cell harvesting in each experiment was conducted in normoxia (for normoxic samples) and in anoxia for hypoxic or anoxic samples whereby the gas taps were locked on the hypoxia chamber after the removal of the hypoxic gas inlet to prevent re-oxygenation of hypoxic samples.

After incubation, 200 μL medium was collected into 1.5 mL Eppendorfs for analysis and the rest was discarded. As all cell lines were adherent they were quickly washed in 3 × 500 μL physiological saline solution (0.85 % NaCl) at room temperature. Subsequently 20 μL distilled H_2O was added and cell lysates were scraped into 1.5 mL Eppendorf tubes.

Prior to use a 96-well silicon plate was rinsed with dH_2O followed by chloroform and ethanol and allowed to dry at room temperature. Leaving the first well blank for acquisition of a reference IR spectrum, 20 μL aliquots of medium or cell sample were evenly applied to the plate and dried at 50 °C for 30 min.

FT-IR analysis was carried out on a Bruker Equinox 55 infrared spectrometer equipped with a motorised microplate module HTS-XT™ utilising a deuterated triglycine sulfate (DTGS) detector (Bruker Ltd.) described in Winder *et al.* (2006)[2] and below. The plate was loaded onto a motorised microplate module from which the plate is introduced into the airtight optics of the instrument where residual moisture is removed by desiccation. The DTGS detector was used to measure transmission throughout the samples, collecting spectra which were displayed in terms of absorbance over a 4000 – 600 cm^{-1} wavelength, as controlled by Opus 5.5 FT-IR software. Each spectrum contained 64 coadds and was acquired in approximately 1 min, and all settings were maintained during all measurements. For any one experiment, samples were analysed in a randomised order.

ASCII data were exported from the OPUS software and imported into Matlab version 9 (The Mathworks, Inc., Natick, MA, USA). Matlab was programmed to replace CO_2 peaks at approximately 2400-2275 cm^{-1} and 680-660 cm^{-1} with a trend from directly before and after the peak in order to reduce any residual atmospheric variation which may affect data processing. Following this, in house routines were programmed to scale the FT-IR spectra using extended multiplicative scatter correction (EMSC)[3].

Validation of HIF functionality

Samples for metabolic profiling by GC-MS were collected over four batches for HCT 116 cells and four batches for HEPA-1 cells, where each batch contained an equal number of WT and HIF-1 deficient cells exposed to normoxia, hypoxia or anoxia. In total there were six experimental groups per cell model (WT and HIF-1 deficient forms exposed to normoxia, hypoxia or anoxia). In every batch, the level of HIF-1 transcriptional activity was measured for a sample in each experimental group using a luciferase reporter-based assay as described previously [4] and below. This was performed as a quality check to ensure HIF-1 transcriptional activity consistency between batches of samples collected for GC-MS analysis.

Two 96-well plates were prepared containing 6000 cells per 200 μl for each cell line in triplicate. All experimental wells were surrounded by wells containing 200 μl medium. Both plates were incubated for 24 h under normoxic conditions. After incubation, all experimental medium was removed and replaced with medium containing HRE luciferase adenovirus at a multiplicity of infection equivalent to 20 viral particles per cell. This adenovirus contains a firefly luciferase reporter construct that is linked to the lactate dehydrogenase HRE sequence as described previously [5]. All reagents were supplied by Promega (Promega corp., Madison, USA) and the analysis was performed in accordance with the manufacturer's guidelines. The plates were then incubated under normoxic conditions for a further 5 h with the virus, after which one plate was transferred to the anoxic condition and both plates were incubated in their conditions for 24 h. Following treatment, all medium was removed from plates and cells were washed twice with ice cold PBS and 50 μL of passive lysis buffer (PLB) solution (1 part PLB in 4 parts distilled water) was added to each experimental well before both plates were shaken in the dark for 15 min. A preset protocol for firefly luciferase on Optima software was used with luciferase assay substrate to measure the luminescence of each well using a MicroLumant LB 96 lumiometer (EG & G Berthold Technologies, Harpenden, U.K.). The degree of luminescence in this case is relative to the expression of HIF-1.

In all cases, HIF-1 was shown to increase in hypoxic and anoxic WT cells compared to normoxic WT cells; a trend that was not observed, or observed to a much lower effect, in HIF-1 deficient cells. The results from this are shown in Figure 1.

GC-MS profiling of cell lysates

From the analysis of both WT and DN cells, a total of 42 and 41 peaks passed our protocol tests and had RSDs lower than 30 % in the GC-MS profiling of QC samples in the HCT 116 and HEPA-1 models respectively. These were identified to MSI guidelines as (referred to in methods) and are listed in the following table. All metabolites were identified in both HCT 116 and HEPA-1 cells except for leucine and xylitol/ribitol which were only identified in HCT 116 cells and phosphocreatinine which was only observed in HEPA-1 cells. The quality of identification is given for each, where putative identifications are explained. Where there were multiple peaks with a single identification these are numbered. Kyoto encyclopedia of genes and genomes (KEGG) compound identifiers are given where available. Heat maps of peak intensities were generated for each model using metaboanalyst software[6] and are shown in Figure 2. Although 41 peaks are present in HEPA-1 data and 42 in HCT 116 data, all 42 peaks have been included in the heat maps so that the peak numbers are comparable between each. Peak 9 did not pass the filters in the HEPA-1 data and was therefore not included in any other analysis, other than in the generation of the heat map. Peak numbers are in accordance to the order in CVA detailed in the manuscript and in table 1 below.

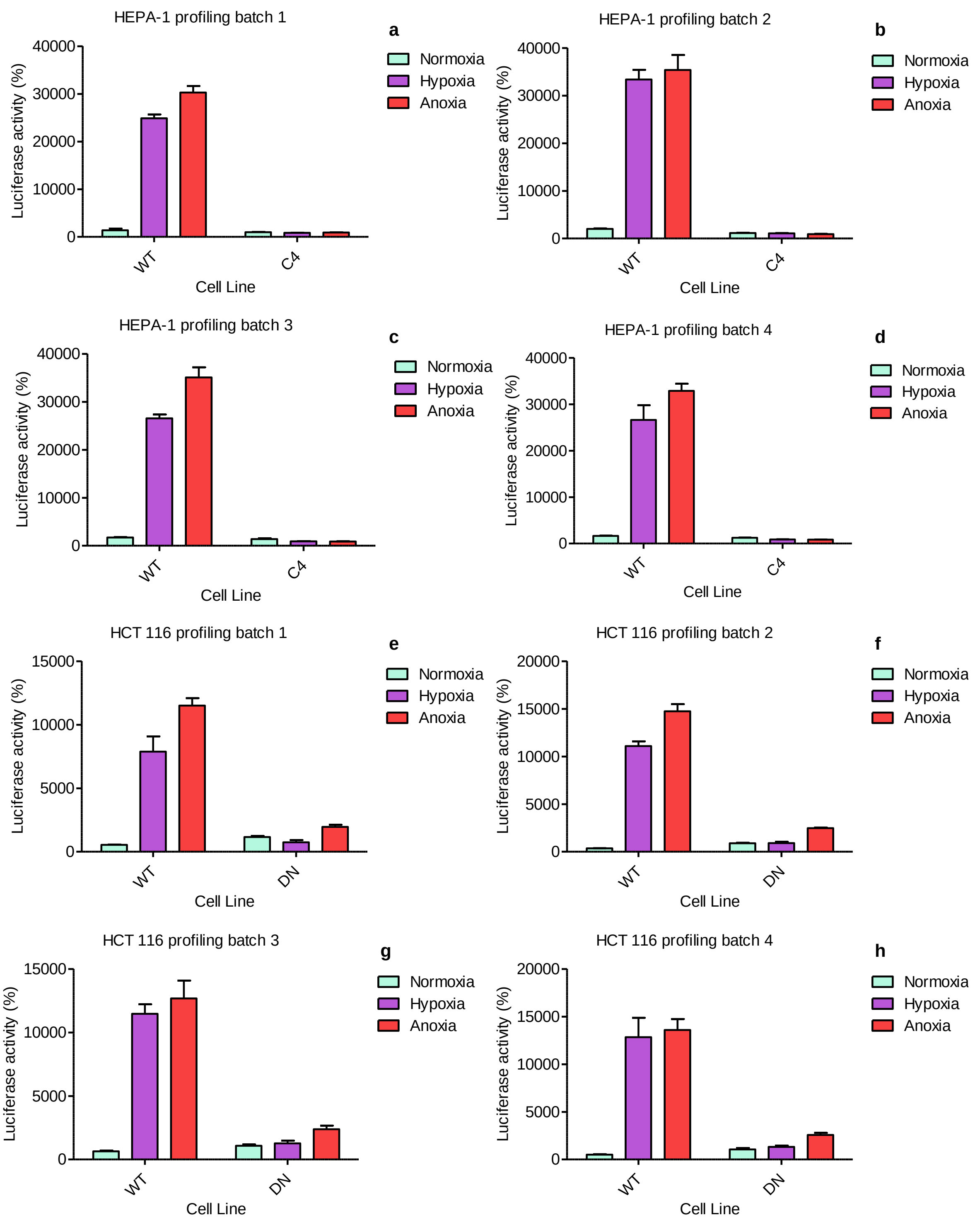

Figure 1: The absolute luciferase activity as a representation of hypoxia inducible factor 1 (HIF-1) activity. The activity was analysed using a luciferase reporter-based assay with an adenovirus containing a firefly luciferase reporter construct linked to the lactate dehydrogenase (LDH) hypoxia response element (HRE) sequence. The luminescence was measured to indicate the degree of HIF-1 expression for HEPA-1 wild type (WT) and C4 cells exposed to normoxia (21 % oxygen), hypoxia (1 % oxygen) and anoxia (0 % oxygen) in each of the four batches of profiling samples (a-d) and for HCT-116 WT and dominant negative (DN) cells exposed to normoxia, hypoxia or anoxia in each of the four batches of profiling samples (e-h).

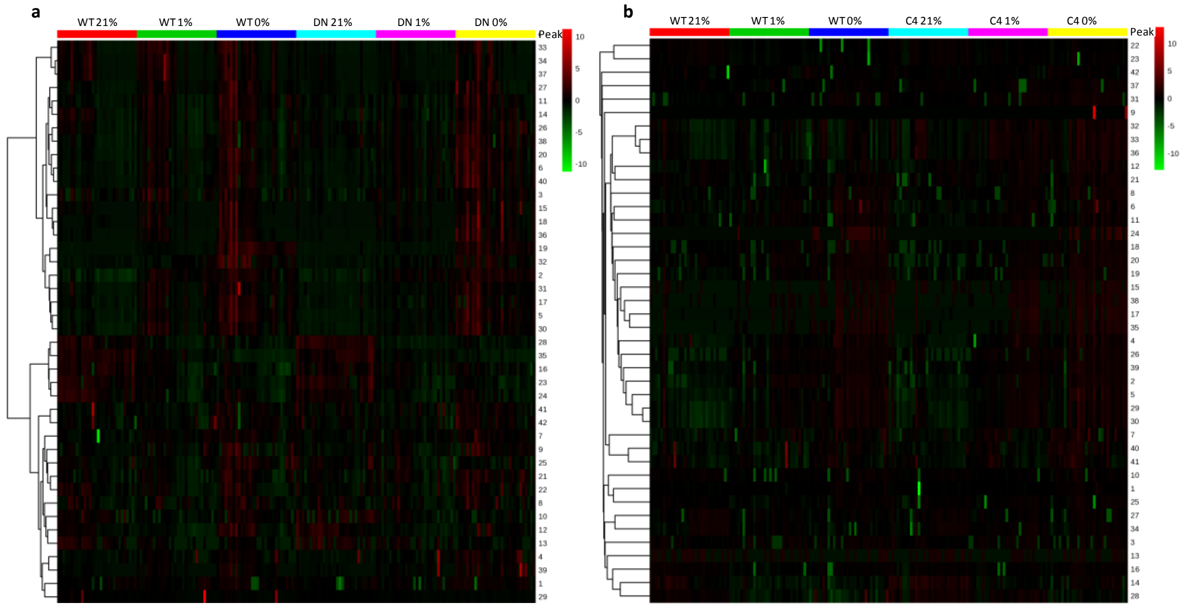

Figure 2: Heat maps of GC-MS peak intensities for a) HCT 116 data and b) HEPA-1 data.

Table 1: Peaks identified in GC-MS analysis of cell lysates

Metabolite peak	Quality of identification	KEGG compound ID
Glycine	High Confidence	C00037
Lactate	High Confidence	C00186
Pyruvate	High Confidence	C00022
Leucine	High Confidence	C00123
Glycerol	High Confidence	C00116
Norleucine	High Confidence	C01933
Malonate	High Confidence	C00383
Phosphate	High Confidence	C00009
Threonine	Putative due to low MS match score	C00188
Phosphocreatinine	High Confidence	
Beta-alanine	High Confidence	C00099
Erythronate/ Threonate	Putative (alternative library match)	C01620
Malate	High Confidence	C00149
4-aminobutyrate	High Confidence	C00334
Aspartate (1)	High Confidence	C00049
Aspartate (2)	High Confidence	C00049
4-hydroxyproline	High Confidence	C01015
Xylitol/ Ribitol	Putative due to RI error	C00379/ C00474
Cysteine	High Confidence	C00097
2-hydroxyglutarate	High Confidence	C02630
Methionine	High Confidence	C00073
Creatinine	Putative due to RI error	C00791
Glutamine	High Confidence	C00064
Putrescine	High Confidence	C00134

Glutamate	Putative due to low MS match score	C00025
Hypotaurine	High Confidence	C00519
Pyroglutamate	High Confidence	C01879
Fructose (1)	Putative due to RI error	C00095
2-oxoglutarate	Putative due to RI error	C00026
Fructose (2)	Putative, due to RI error	C00095
Fructose / Sorbose (1)	Putative due to RI error and identical EI mass spectra	C00095/C00764
Fructose / Sorbose (2)	Putative due to RI error and identical EI mass spectra	C00095/C00764
Glycerol-3-phosphate	Putative,due to RI error	C00093
Allose/Mannose/ Galactose /Glucose (1)	Putative due to RI error and identical EI mass spectra	C01487/C00159/ C00124/C00031
Allose/Mannose/ Galactose /Glucose (2)	Putative due to RI error and identical EI mass spectra	C01487/C00159/C00124/C00031
Citrate	Putative due to RI error	C00158
Lysine	High Confidence	C00047
Allose/Mannose/ Galactose /Glucose (3)	Putative due to RI error and identical EI mass spectra	C01487/C00159/C00124/C00031
Scyllo/*Myo*-inositol	Putative due to RI error and identical EI mass spectra	C06153/C00137
Tyrosine	Putative due to RI error	C00082
Tyramine/ Tyrosine	Putative due to RI error and identical EI mass spectra	C00483/ C00082
Hexadecanoic acid	High Confidence	C00249
Octadecenoic acid	High Confidence	C00712

References

1 Kotze, H. L. et al. A novel untargeted metabolomics correlation-based network analysis incorporating human metabolic reconstructions. Bmc Systems Biology 7, doi:107 10.1186/1752-0509-7-107 (2013).

2 Winder, C. L., Gordon, S. V., Dale, J., Hewinson, R. G. & Goodacre, R. Metabolic fingerprints of Mycobacterium bovis cluster with molecular type: implications for genotype-phenotype links. Microbiology-Sgm 152, 2757-2765, doi:10.1099/mic.0.28986-0 (2006).

3 Martens, H., Nielsen, J. P. & Engelsen, S. B. Light scattering and light absorbance separated by extended multiplicative signal correction. Application to near-infrared transmission analysis of powder mixtures. Anal. Chem. 75, 394-404, doi:10.1021/ac020194w (2003).

4 Burrows, N. et al. Expression of hypoxia-inducible factor 1 alpha in thyroid carcinomas. Endocrine-Related Cancer 17, 61-72, doi:10.1677/erc-08-0251.

5 Cowen, R. L. et al. Hypoxia targeted gene therapy to increase the efficacy of tirapazamine as an adjuvant to radiotherapy: Reversing tumor radioresistance and effecting cure. Cancer Res. 64, 1396-1402 (2004).

6 Xia, J., Sinelnikov, I. V., Han, B. & Wishart, D. S. MetaboAnalyst 3.0—making metabolomics more meaningful. Nucleic Acids Res., doi:10.1093/nar/gkv380 (2015).
